# Programmable electronic synapse and nonvolatile resistive switches using MoS_2_ quantum dots

**DOI:** 10.1038/s41598-020-68822-5

**Published:** 2020-07-24

**Authors:** Anna Thomas, A. N. Resmi, Akash Ganguly, K. B. Jinesh

**Affiliations:** Department of Physics, Indian Institute of Space-Science and Technology (IIST), Valiyamala, Thiruvananthapuram, 695547 Kerala India

**Keywords:** Materials for devices, Nanoscale materials, Electronics, photonics and device physics

## Abstract

Brain-inspired computation that mimics the coordinated functioning of neural networks through multitudes of synaptic connections is deemed to be the future of computation to overcome the classical von Neumann bottleneck. The future artificial intelligence circuits require scalable electronic synapse (e-synapses) with very high bit densities and operational speeds. In this respect, nanostructures of two-dimensional materials serve the purpose and offer the scalability of the devices in lateral and vertical dimensions. In this work, we report the nonvolatile bipolar resistive switching and neuromorphic behavior of molybdenum disulfide (MoS_2_) quantum dots (QD) synthesized using liquid-phase exfoliation method. The ReRAM devices exhibit good resistive switching with an On–Off ratio of 10^4^, with excellent endurance and data retention at a smaller read voltage as compared to the existing MoS_2_ based memory devices. Besides, we have demonstrated the e-synapse based on MoS_2_ QD. Similar to our biological synapse, Paired Pulse Facilitation / Depression of short-term memory has been observed in these MoS_2_ QD based e-synapse devices. This work suggests that MoS_2_ QD has potential applications in ultra-high-density storage as well as artificial intelligence circuitry in a cost-effective way.

## Introduction

The computers we are using at present are based on the von Neumann architecture^1^, in which a processor is connected to the memory through a bus. The processor speed has increased significantly in recent years, but at the same time, the memory or data storage capability also has been tremendously increased by the advent of new technologies. However, the data transfer rates remain small, which limits the speed and overall computational capabilities. This inherent limit imposed by the computational architecture is called von Neumann bottleneck, and it is obvious that establishing alternative approaches is essential to cope up with the computational speeds required for future technologies. One such promising alternative is the computation based on artificial neural networks, which mimics how the biological brain functions. The parallel processing and learning capacity of the biological brain arise from the multitude of synaptic connections of individual neurons with tens of thousands of other neurons. The essential building blocks of brain-inspired technology, known as neuromorphic computation, are artificial synapse, devices that mimic the functions of biological neurons. The synaptic weight is modulated in an analogue manner by various ion dynamics through the synaptic terminals during the learning process^2^. The mechanisms of neural networking have been successfully reproduced using various device configurations, such as capacitors and transistors, and has opened up a vast possibility of realizing brain-inspired computation. Thus, artificial synaptic systems inspired by the unique properties of the biological neural systems offer the possibilities of parallel computations with low power consumption and self- learning ability and have the potential to overcome the von Neumann bottleneck, the classical complementary metal–oxide–semiconductor (CMOS) technology currently faces^3^.

The strength of a biological learning process is measured as synaptic weight, which is the strength of a synapse acquired by the repeated ionic exchange through the synaptic cleft as a result of action potentials. In electronic synapse (e-synapse), the way to measure synaptic strength is by monitoring the variation in the conductivity of a two-terminal (resistor or capacitor like) device or by monitoring the threshold voltage of a transistor-like three-terminal device. Ideally, a memristor device resembles a biological synapse and can manifest a variety of functions of the biological neural system. The synaptic weight in this case is the measure of conductance of a memristor upon applying voltage pulses that resemble the action potentials. For a memristor to perform as an artificial synapse, it should have the capability to vary its charging capacity when modulated by a voltage pulse. Interestingly, a large class of nanomaterials has been investigated to understand their neuromorphic responses, because nanostructures have a large number of surface defects that can be electrically modulated^4–7^.

Another ingredient to develop artificial neural networks is the large memory density in par with the biological memory but in a miniaturized form. For that, resistive memory technology, the precursor for neuromorphic computation, has attracted a great deal of attention as nonvolatile memory (NVM) due to its scalability, high operational speed, and CMOS compatibility^8,9,10^. The resistance switching behavior has been reported for a variety of materials such as perovskite-type oxides, binary metal oxides, solid-state electrolytes, organic compounds, amorphous Si^11^ and semiconducting chalcogenides^12^. Among these materials low dimensional materials such as two-dimensional materials (2D), one-dimensional materials (1D), and zero-dimensional materials (0D) offer wide possibilities of stoichiometry, defect engineering, and interfacial chemistry, which underlie synaptic behavior in general^13^. Moreover, the mechanical flexibility of low dimensional nanomaterials facilitates the development of flexible/wearable neuromorphic device applications^14,15^. Recently, promising neuromorphic functions have been observed in hybrid systems, which include low dimensional semiconducting chalcogenide materials^14,16,17^. Semiconductor chalcogenides have been extensively studied for memory applications^18–20^. Several interesting works have been reported so far in this direction; for example, monolayer transition metal dichalcogenides (TMDCs) of the formMX_2_ (*M* = Mo, W; *X* = S, Se) sandwiched between metal electrodes form an ultrathin vertical memristor (thickness < 1 nm), whose low On-state resistance (< 10Ω) enables high-frequency switches that operate at 50 GHz^21,22^. Their high switching On–Off ratio (> 10^4^) refutes the problem of leakage currents in monolayer semiconductors while scaling down the nonvolatile memory device to the sub-nanometer scale, suggesting new switching mechanisms in which, point defects are likely playing a central role^13,21,22^. Recently an innovative idea of memory capacitor switches for inexpensive printed electronic applications has been introduced by Bessonov et.al. using MoS_2_ based devices. The device has programming voltage from 0.1 to 0.2 V and an adjustable resistance of 10^2^–10^8^ Ω. Due to the nonlinearity of switch dynamics, different synaptic flexibility is achieved through a series of electric pulses^23^. Recently, the nonvolatile memory devices based on nanoparticles or quantum dot of various materials have been studied extensively ^24–26^. Further, a memory concept based on self-organized quantum dots (QD), with a write time of 6 ns has been introduced. The physical limitation of the write time of such a QD-based memory known to be in the picosecond range^27^. Among the existing nanomaterials, MoS_2_ nanostructures have attracted recent interest due to their simple stoichiometry and good switching characteristics^28^. Several combinations of MoS_2_ have been reported for memory applications, which include MoS_2_-graphene ultra-thin stack that produces a hysteresis in the transistor behavior due to charge trapping, MoS_2_ embedded in polymethyl methacrylate (PMMA) matrix, which exhibits quantum conductance due to Coulomb blockade effect etc^29^.

MoS_2_ is a promising material for most of the electronic applications since monolayer MoS_2_ has a direct bandgap with large conductivity and electron mobility, in addition to its potential as a promising material for flexible electronics applications. However, due to the large electrical conductivity, it may not be suitable for resistive switching applications as such, since the Off-current will be high and the On–Off ratio will be small. However, this problem can be circumvented by reducing its dimensions to a few nanometers and thereby increasing its bandgap. MoS_2_ has a large exciton Bohr radius of 23 nm^30^, which facilitates easily achievable size quantization. Also, the synthesis of MoS_2_ quantum dots can be a simple one-step exfoliation process.

In this article, we report the nonvolatile memristive behavior and the neuromorphic properties of liquid-phase exfoliated MoS_2_ quantum dots (QD), prepared by a simple one-step exfoliation process. Using thin films comprising MoS_2_ QD, we fabricated two types of devices, one is resistive random access memory (ReRAM) devices and second is neuromorphic devices employing QD layers of different thickness and different top electrodes. ReRAMS were fabricated with 200 nm MoS_2_ QD layers in FTO/MoS_2_ QD/Al device configuration, while neuromorphic devices were fabricated using 400 nm thick MoS_2_ QD layers in FTO/MoS_2_ QD/Au device configuration. We demonstrate excellent resistive switching in FTO/MoS_2_ QD/Al devices and consistent retention and cycling properties. In the neuromorphic configuration, the MoS_2_ QD e-synapse exhibits Short Term Potentiation (STP), shown by measuring the Excitatory Post-Synaptic Current (EPSC) of FTO/MoS_2_ QD/Au devices. This e-synaptic behavior is achieved by the charge trapping and de-trapping in the quantum dots by applying electric pulses, which resemble action potentials in the biological brain. As we demonstrate in this manuscript, MoS_2_ QDs act as excellent candidates for e-synapse, and their synaptic responses such as paired-pulse facilitation (PPF) and depression (PPD) have been studied here as a function of various control parameters such as duty cycle, On and Off duration and frequency of the action potentials.

## Results and discussions

### Material characterization

**Figure 1:**
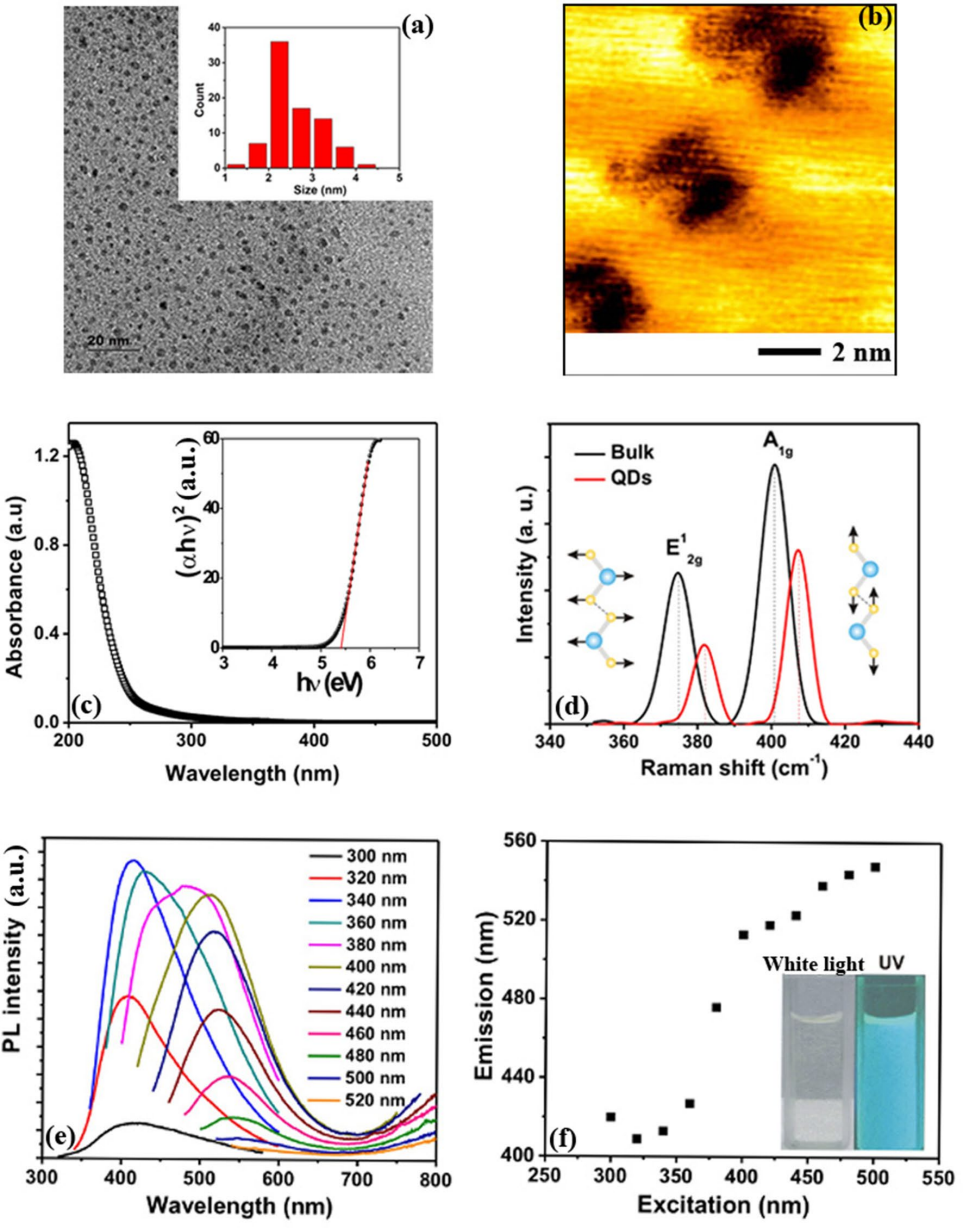
Morphology of QDs. (**a**) Transmission electron microscopic images of MoS_2_ QDs. The inset shows the statistical size distribution of QDs from the transmission electron microscopy (TEM). (**b**) STM images of MoS_2_ QDs on a HOPG surface. The image has been taken in constant current mode at a bias voltage of − 0.5 V and 0.5 nA current. (**c**) The UV–Visible absorption spectrum of the MoS_2_ QDs; the inset shows the Tauc plot assuming a direct bandgap material. (**d**) Raman spectra MoS_2_ bulk and QDs, showing the in-plane ($E_{2g}^{1}$) and out-of-plane ($A_{1g}^{{}}$) vibrational modes of the S atoms. The blue shift of the peak for QDs is due to quantum size effects in MoS_2_. (**e**) Photoluminescence (PL) spectra of the aqueous solution of MoS_2_ quantum dots for different excitation wavelengths; (**f**) the dependence of emission wavelengths on excitation wavelengths. The inset shows the quantum dot suspension in water under white light and its blue fluorescence under UV irradiation.

Different microscopic analyses have been carried out to understand the distribution and morphology of the QDs. Transmission electron microscope (TEM) images of the MoS_2_ quantum dots are shown in Fig. 1a. The average diameter of the MoS_2_ QD is 2.5 nm from the statistical size distribution of particle sizes. Scanning tunneling Microscopic (STM) analysis of the QDs has been done by dispersing the QD solution on highly oriented pyrolytic graphite (HOPG) substrate. In Fig. 1b, the bright region represents HOPG and the darker regions represent the quantum dots. The size of the QD observed from TEM and STM are matching very well. Ultraviolet–visible (UV–VIS) absorption spectrum (Fig. 1c) of MoS_2_ QD shows a strong blue shift and an absorption peak located around 206 nm. From the Tauc plot, a bandgap of 5.1eVcan be measured for MoS_2_ QD, which indicates strong quantum size effects in the samples, compared to the bulk MoS_2_^31,32^.

Figure 1d shows the Raman spectra of the MoS_2_ quantum dots. Raman spectra exhibit two distinguishable peaks that originate from optical phonon modes $E_{2g}^{1}$ and $A_{1g}^{{}}$ corresponding to in-plane and out-of-plane vibration of S–Mo–S bonds respectively^33^. Compared to the bulk MoS_2_, both $E_{2g}^{1}$ and A_1g_ peaks are blue-shifted from 374.17 cm^−1^ and 400.43 cm^−1^ to 381.63 cm^−1^ and 407.92 cm^−1^ respectively due to the quantum confinement effect of MoS_2_ QDs, which is very consistent with the values reported for MoS_2_ nanodots^34^. The blue shift of both in-plane ($E_{2g}^{1}$) and out-of-plane ($A_{1g}^{{}}$) vibrational modes in quantum dots are different from the shift of the corresponding peaks when MoS_2_ changes from multilayers to monolayer, where $E_{2g}^{1}$ mode has a blue-shift and $A_{1g}^{{}}$ has a red-shift ^35,36^.

Similarly, the photoluminescence spectra (PL) of MoS_2_ QDs also shows blue-shifts due to strong quantum confinements. Due to quantum confinement effects, QDs emit different wavelengths when irradiated with an excitation beam^37^. As observed in Fig. 1e, f, the emission spectra are excitation dependent; with the increasing excitation wavelength, a redshift in the emission spectrum can be observed. Generally, MoS_2_ quantum dots exhibit excitation dependent PL spectra due to the heterogeneous distribution of the particle sizes and polydispersity of the dots ^38^.

### Resistive Random-Access Memory (ReRAM) device fabrication and characterization

**Figure 2:**
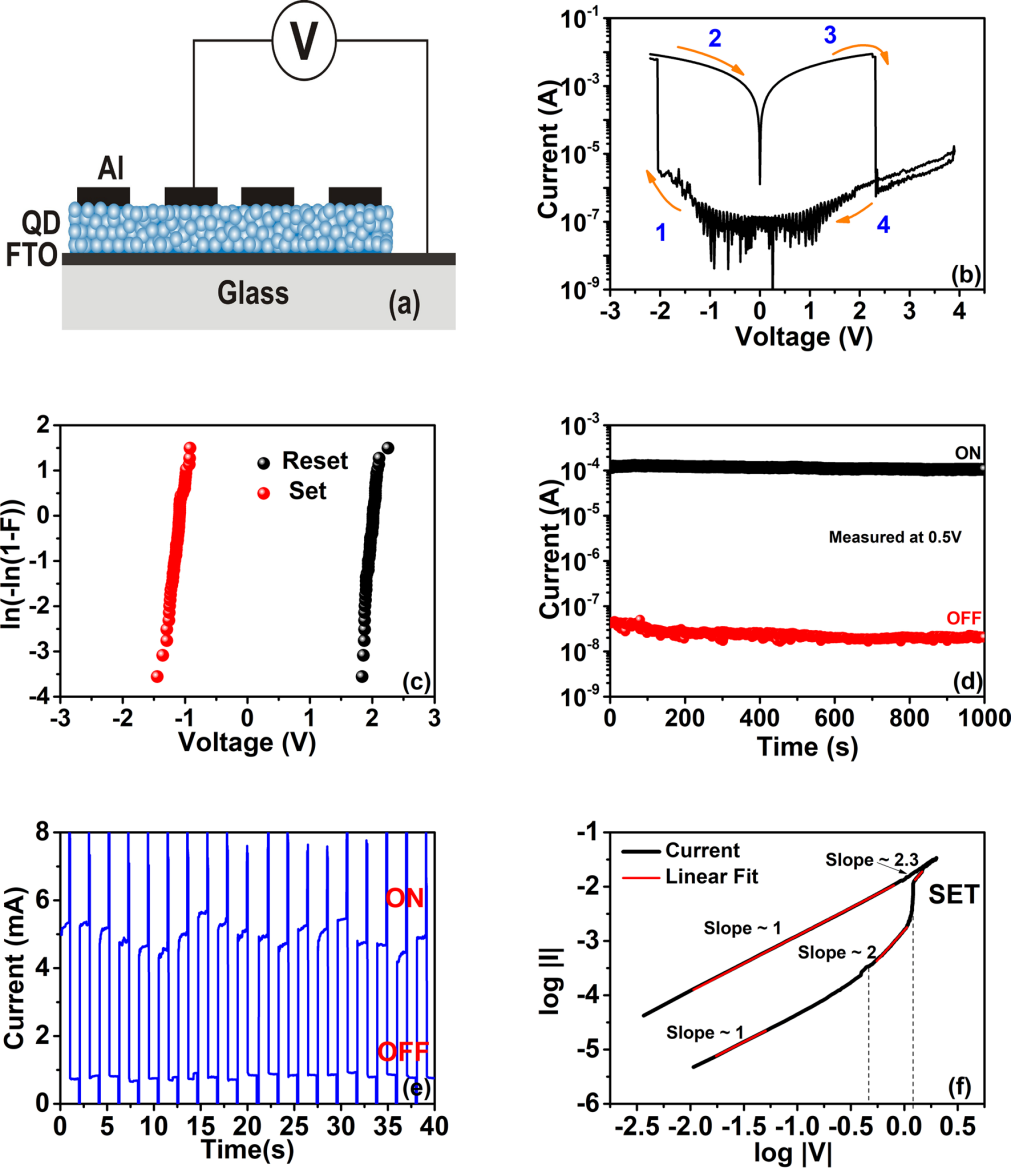
MoS_2_ QD based resistive switching memory device: (**a**) schematic representation of the memristive device in a configuration FTO/MoS_2_/Al; (**b**) *I–V* characteristics of MoS_2_ memory device in which SET and RESET process around − 2 V and 2 V respectively; (**c**) Weibull plots for SET and RESET process in the device; (**d**) Retention measurement of the device at a constant voltage of 0.5 V, from which the On–Off ratio is observed to be around 10^4^, the device is stable up to 1000 s. (**e**) Cycling measurement in a sequence of write–read–erase–read with voltage pulses of − 1.9, 0.5, 2.2, and 0.5 V respectively. The data measured for 2000 s is shown in S2. (**f**) Analysis of the conduction mechanism in the memristive device, which depicts the Ohmic and space charge limited conduction (SCLC).

Details of the fabrication of the memristive devices are described in the experimental section. The device configuration used for ReRAM characterization was fluorine-doped tin oxide/MoS_2_ QD/aluminium (FTO/MoS_2_ QD/Al), as shown in Fig. 2a. The current–voltage (*I–V*) characteristics of the device are shown with arrow marks to depict the sequence of the measurements (see Fig. 2b). The bias voltage was swept from − 2 to 3 V and reverse. The arrow 1 shows the initial sweep (0 V to − 2 V), which shows that the device is initially in the high resistive state (HRS or Off state). At around − 2 V the device suddenly changes its state from HRS to low resistance state (LRS). The device continues to be in the On state during the voltage sweep from − 2 V to 0 V and 0 V to 3 V indicated by arrows 2 and 3 respectively. Thereafter, the device changes from LRS to HRS (RESET process) as indicated by arrow 4. After the RESET, the device continues to be in the Off state until the next SET process.

The mean RESET voltage of the ReRAM devices is 1.98 ± 0.08 V and SET is − 1.12 ± 0.11 V. The operational consistency of the devices could be evaluated from the cumulative SET and RESET data plotted as Weibull distributions^39^. The Weibull plot in Fig. 2c has a single slope without anomalies, which indicates a highly reliable switching process. The stability of the device has been measured by evaluating the current by applying a constant voltage in Off state as well as in On state. The retention measurements^40^ has been carried out for 1000 s at a constant voltage of 0.5 V at On state and then at Off state, and are shown in Fig. 2d. Generally, we observed an On–Off ratio of 1,000, though some devices exhibited the On–Off ratio as high as 10^4^. We performed write-erase cycling and observed for 60 repeated switching cycles, the I–V curves of the devices have no apparent degradation (see figure S1). Write–read–erase–read sequential measurements were performed in ambient conditions to confirm the switching characteristics of the device. The write, read, and erasing processes of the device were performed at pulse voltages of − 1.9, 0.5, and 2.2 V respectively. This measurement has been carried out for 2000s and is shown in figure S2. It is noted that the read voltage for our device is lower as compared to the existing reports related to the MoS_2_ nanoparticle-based memory^41,42^.

Further investigation has been made to understand the underlying conduction mechanism in the device. Generally, the device will be in the high resistance state (HRS or Off state). In contrast, the MoS_2_ QD based memristor is initially in Low Resistance State (LRS or On state). This is presumably due to sulphur vacancies on the surface of MoS_2_, which act as electron donors and induce localized states in the bandgap. The charge transport is prompted by hopping through defect induced localized states, which constitute the initial low resistance state^43^. Since the defect formation energy for the sulphur vacancy (V_S_) is lower as compared to the molybdenum vacancy (V_Mo_), the former is more likely to happen during the exfoliation process^44^. Afterward, these filaments get broken due to the thermal stress of the defects as a result of the Joule heating, subsequently resetting to HRS (OFF state).

The log–log plot of the *I–V* curve during the SET process is shown in Fig. 2f. Followed by the Ohmic region, the presence of V^2^ dependence of the current indicates that the conduction mechanism in HRS is dominated by space charge limited conduction (SCLC)^45^. The SCLC regime arises due to charge trapping and de-trapping at the interfaces between MOS_2_ QDs, Al/MoS_2_ interface, or FTO/MoS_2_ interface. The initial conduction is due to the thermally generated free charge carriers or trapped charges that are free to conduct on applying the electric field. Therefore, initially it follows Ohm’s law, which is represented in the initial part of the curve in Fig. 2f with a slope 1. At higher applied voltages the conduction is through the SCLC mechanism and is governed by Child’s law $J = \frac{9}{8 }\mu \varepsilon \frac{{V^{2} }}{{d^{3} }}$ , where µ is the carrier mobility, ε the dielectric permittivity of the active medium and *d* is the thickness of the film^45^. The voltage at which the Ohm’s low regime changes to the SCLC regime is called transition voltage (V_Tr_). In our device, this transition voltage was measured around 0.5 V. The trap-filled regime is appearing around 1.2 V (V_TFL_). Followed by the trap-filled regime, the devices switch to On state due to the formation of a complete filament. Even after switching, due to the applied voltage, the injected electrons are captured by the defects and fill the traps. It is clear from the plot that resistance also changing after the SCLC regime, the slope is changing from 2 to 2.3. Ohmic conduction remains after reverting the voltage indicates that the filament remains between two electrodes.

### Neuromorphic device fabrication and characterization

In a real neural network, the transmission of a signal between two neurons occurs via synaptic connections. When an action potential arrives at the axon of a pre-synaptic neuron, neurotransmitters are released from the pre-synaptic neuron through the cell membrane to the synaptic cleft. These neurotransmitters will go through the synapse and activate the receptors in the postsynaptic neuron, further trigger a subsequent action potential in the post neuron. This transmission of action potential produces an excitatory post-synaptic current (EPSC)^46^. Similar to the biological synapse we can make use of a two-terminal memristor as an artificial e- synapse^47^. Here the top and bottom electrodes (FTO and Gold (Au)) act as the pre and postsynaptic neuron, MoS_2_ QD layer act as the synapse. According to the conductivity of the MoS_2_ QD layer, we can modulate the synaptic weight.

Neuromorphic devices were fabricated by spray coating the dispersion of MoS_2_ QDs over an FTO coated glass at 100 °C to obtain a thickness of approximately 400 nm. It is to be noted that we have used gold electrodes instead of aluminum (Al) compared to the previous section. Using aluminum as the top electrode, we haven’t observed any synaptic behavior in the device, the reason behind is likely the Ohmic contact between aluminum (φ_Al_ ~ 4.25)^48^ and MoS_2_ QDs. Gold electrodes were thermally evaporated through a shadow mask of electrode area 1 mm^2^ to create a metal–semiconductor–metal structure. The electrical characterizations of MoS_2_ QD synaptic devices were performed using an Agilent B2900A source measurement unit. Throughout the measurements the bottom electrode FTO was kept grounded, and the voltage was swept on the top Au electrode.

**Figure 3:**
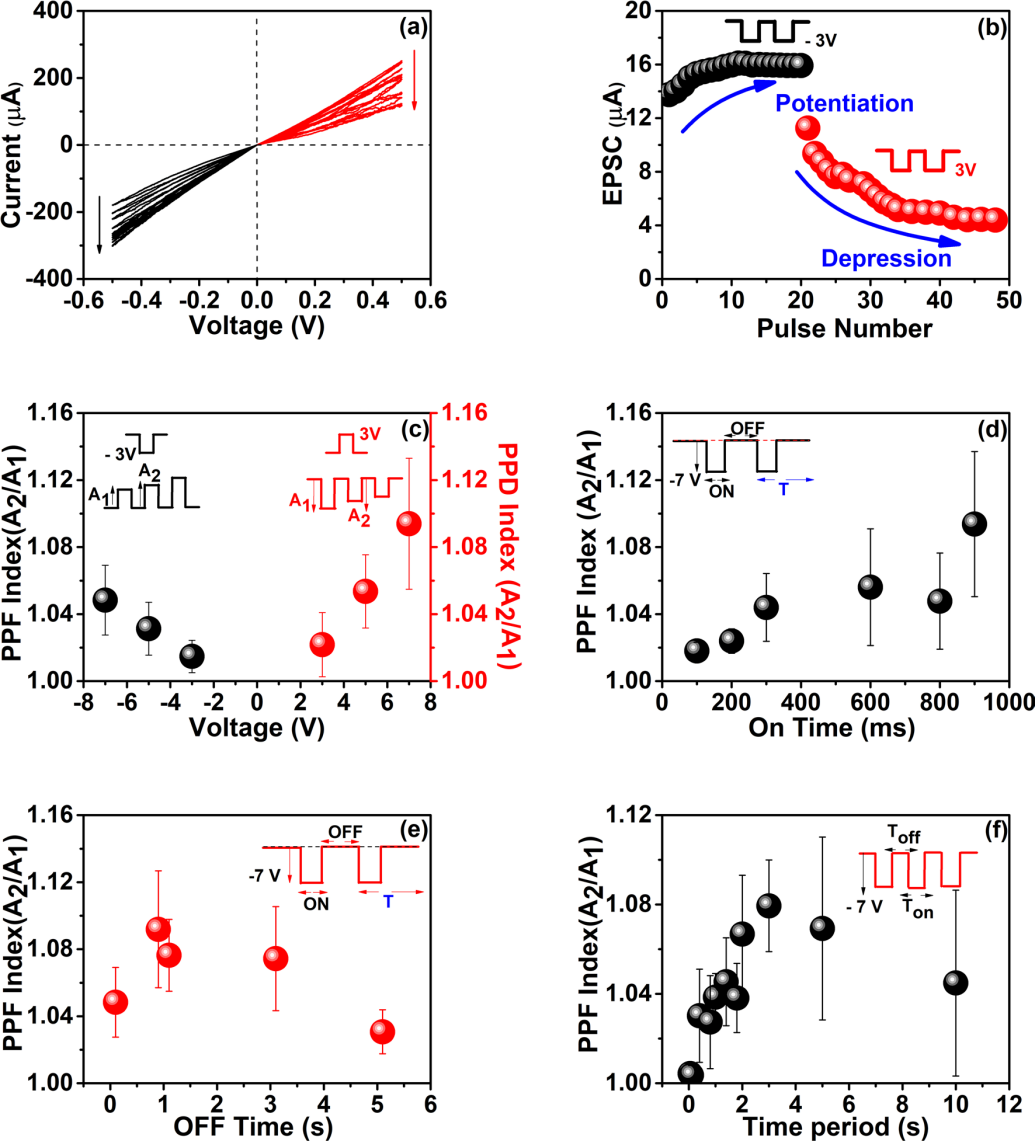
Electrical properties of e-synapse. (**a**) *I–V* characteristics under positive (red lines) and negative voltage sweeps (black lines). (**b**) Excitatory Post-Synaptic Current (EPSC) measured for the FTO/MoS_2_ QD/Au devices. Under negative voltage pulse, it shows potentiation (black spheres) and under positive voltage pulses, it shows depression (red spheres). The inset shows the schematic of the input pulse of 3 V (red line) and − 3 V (black line). (**c**) PPD and PPF variation for the amplitude of the voltage pulse. The left inset shows the schematic of the input pulse of -3 V (black line) and the response of the output current with the second response is larger than the first (A_2_ > A_1_). The right inset shows the schematic of the input pulse of 3 V (red line) and the response of the output current with the second response is smaller than the first (A_2_ < A_1_). (**d**) Dependence of pulse time variations in PPF. Inset shows the input pulse with − 7 V with fixed 900 ms Off time (black line). (**e**) Dependence of off time variations in PPF. Inset shows the input pulse with − 7 V with fixed 900 ms Ontime (red line). (**f**) Dependence of time/frequency in PPF and it shows a band-pass filter characteristic. The inset shows the input pulse with − 7 V (red line).

Figure 3a shows the consecutive *I–V* measurements of the MoS_2_ synaptic devices. Initially, similar to the memory device described earlier, the synaptic device is in the LRS state. The current through the device decreases continuously and saturates at a certain current level when consecutive positive voltage sweeps are applied through the top electrode. As a result, the devices shift to HRS state. Followed by the above phenomenon, the current through the e-synapse increases when consecutive negative voltage sweeps are applied at the gold electrode. The observed gradual change in the conduction state of the QD layer can be either, due to the gradual formation and rupture of several conduction filaments, or due to the charge trapping and de-trapping in the active layer. In other words, we can say that the resistance state of the device gradually changes from low resistance to high resistance or vice versa according to the polarity of the voltage applied at the top electrode. Thus, according to the number of sweeps, one can modulate the conductance, which is similar to the variable synaptic weight.

Subsequently, we examined the synaptic weight as a function of the action potential sequence^49,50^. The conductance of the MoS_2_ QD layer was thus modulated by applying a series of voltage pulses. A series of consecutive voltage pulses with an amplitude of − 3 V, 900 ms On time, and 100 ms Off time (the total period of 1 s) was applied to the device. The observed synaptic response as output current is shown in Fig. 3b. Postsynaptic current gradually increases and saturates at a certain current level. After reaching saturation, a series of pulses with + 3 V and same timescales as above were applied and the resultant postsynaptic current is recorded. Figure 3b shows a clear increasing depression as the number of pulses increase. The postsynaptic current has the same trend as we measure the device by applying the DC sweeps as well as by applying voltage pulses. Thus, the negative voltage pulses potentiate the conductivity and the positive voltage pulses depress the conductivity. Potentiation and depression are the key features of short-term plasticity in a biological synapse. Paired pulse facilitation (PPF)/depression (PPD) is a dynamic reduction/ enhancement of neurotransmitter release, it is considered to be an essential key to transmit information in the biological synapse. PPF index is measured as the ratio of the postsynaptic response to the second presynaptic spike/pulse over the response to the first spike/pulse^46,51^. In our synaptic device, we have demonstrated PPF/PPD modulation by the different amplitudes of voltage pulse (900 ms pulse On time, 1 s period) as shown in Fig. 3c. It is observed that the potentiation increases with the increase in the amplitude of the voltage pulse, and the PPD index keeps on increasing with the increase in the amplitude of the voltage pulse in the positive direction. The amplitude dependence of the PPF indicates that it originates from the manipulation of charges through defects; indicating a mechanism where the conductivity of the charge transport paths varies with charge trapping. This results in achieving modulation of synaptic function or in other words, the gradual change in the conductivity resembles a successively changing synaptic weight. Yan et al. have recently reported that electron hopping between sulfur and metal vacancies is the reason for ultra-fast memristive behavior in two-dimensional metal chalcogenide films.^16,23,52,53^ The charge hopping is voltage-dependent, which leads to voltage-dependent PPF as observed in Fig. 3c. We believe that the same is the underlying mechanism behind observing the PPF/PPD in MoS_2_ QD systems as well, and has been discussed in detail later on in this work.

In addition to the amplitude of the pulse train, another three factors also can modify the facilitation in any synaptic device. They are frequency of the input pulse, the time interval between two consecutive pulses (Δ*t*), and the pulse time. A detailed study of the effect of aforesaid factors in our synaptic device is shown in Fig. 3d–f. Figure 3d demonstrates that the PPF index keeps on increasing with the increase in pulse time with constant 900 ms Off time. Thus, we understand that while increasing the pulse time, the QDs get sufficient time to de-trap the charges while increasing the pulse time (On time), resulting in increasing PPF. Similarly, the amplitude of the output, or the learning process, caused by the second pulse is determined by the time interval between two pulses, larger the interval smaller will be the change in amplitude. This replicates the exact learning process that happens through neurons in the biological brain, where the frequency of the neural signals decides the synaptic weight. In this e-synapse, PPF keeps on decreasing with the increase in Off time (Δt). Within 5 s Off time, it is observed that facilitation almost dying out or there is no amplitude change in output current.

For a synapse, the facilitation builds up and decays within a time interval, which can be approximated as a double exponential function given below^2^.

1 $$ A_{2} /A_{1} = C_{1} \times exp^{{\left( {\frac{{ -\Delta t}}{{t_{1} }}} \right)}} + C_{2} \times exp^{{\left( {\frac{{ -\Delta t}}{{t_{2} }}} \right)}} + C_{0} , $$

where A_2_ and A_1_ are the amplitude of the postsynaptic current with respect to the second and first strike of voltage pulses. Δ*t* is the time interval between pairs of consecutive pulse signals, *C*_1_ and *C*_2_ are the initial magnitudes of the fast and slow phases, the relaxation times of the fast and slow phases are represented by *t*_1_ and *t*_2_, respectively. From this approximation, we can conclude that, while increasing Δ*t*, we are giving the system enough time to relax to its original conduction state. As a result, the facilitation exponentially decays. That is, the e-synapse is more likely to remember the information with an Off time of 1000 ms. The frequency dependence has been studied with a frequency window of 0.1 Hz to 20 Hz in our e-synapse and is shown in Fig. 3f. In this device, the PPF index keeps on increasing with increasing frequency. Maximum PPF was obtained at 0.33 Hz (period of 3 s). Further increase in frequency causes a reduction in the facilitation of conductivity. It can be concluded from the frequency response of the PPF index that the maximum remembering efficiency of these e–synaptic devices is obtained when the frequency of the action potential is 0.33 Hz. Also, this experiment reveals the ability of the MoS_2_ QD synaptic device to function as a low-band pass filter, because, at lower frequencies, the device gets enough time to relax, and at higher frequencies, the trapped electrons are less responsive to the input pulses^5^.

**Figure 4:**
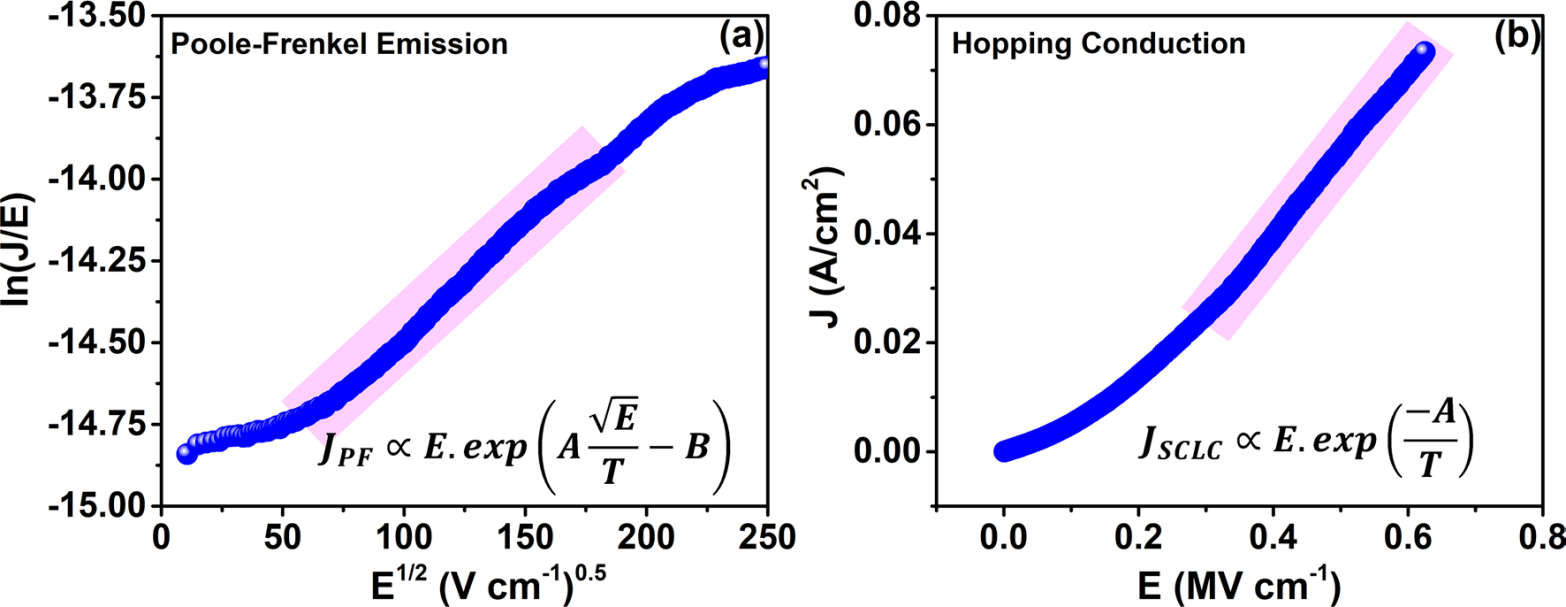
Analysis of underlying conduction mechanism. (**a**) ln(*J*/*E*)–*E*^1/2^ plot to identify the Poole–Frenkel mechanism. (**b**) J–E plot for analyzing the hopping conduction in the synaptic device. The highlighted regions represent the voltage regime, in which the proposed conduction mechanisms happen.

To understand the physics behind the neuromorphic nature of the MoS_2_ QD synaptic devices, the *I–V* characteristics have been studied extensively to identify the responsible conduction mechanisms. MoS_2_ QDs have a large number of surface defect densities, primarily because of the sulfur and molybdenum vacancies that arise during the liquid-phase exfoliation process^54^. In addition, quantum dots itself have charge storage capacity similar to a capacitor^5^. These defects levels involve in continuous charge trapping and release with characteristic timescales. In these devices, these characteristic timescales vary from a few milliseconds to several seconds. At low voltages, these trapped charges would form space charges, leading to space charge limited currents (SCLC) through the device. Figure 4 shows the analysis of different bulk limited conduction mechanisms in our devices. The conduction through the switching medium is a combination of Poole–Frenkel charge injection, inter-trap charge hopping and SCLC^45^. Figure 4a is a plot between ln(J/E) and E^1/2^, highlighting the voltage regimes at which Poole–Frenkel emission occurs through the layer.

**Figure 5:**
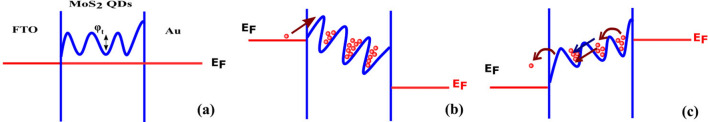
Schematic band diagram of our e-synapse. (**a**) Under equilibrium, a Schottky barrier is developed at the Au/MoS_2_ interface and there are traps with trap height ϕ_t_ in the MoS_2_ QD layer. (**b**) Trapping of electrons in the defects under positive pulses. (**c**) De-trapping of electrons via hopping and Poole–Frenkel emission under negative pulses.

Similarly, the inter-trap hopping mechanism is the tunneling of the trapped electrons from one trap to another. Figure 4b shows the ln(J)–ln(E) plot to identify hopping conduction through the device under the applied bias. The linear relationship between J and E indicates that hopping through traps is one of the main reasons behind the electron trapping and de-trapping, leading to e-synaptic behavior. Figure 5 shows the representative band-diagram of the FTO/MoS_2_ QD/Au devices. The work function of MoS_2_was estimated using scanning tunneling spectroscopy and was found to be approximately 5.2 eV (The details of the calculation and the *I-z* spectrum can be seen in supporting information; figure S3). The standard values of work function for gold ($\varphi_{Au} = 5.4\,{\text{eV}}$)^55^ and FTO ($\varphi_{FTO} = 4.4\,{\text{eV}}$)^56^ have been adapted from earlier reports. From Fig. 5, we can understand that the conduction band bends up at the Au/MoS_2_ interface and bends down at FTO/MoS_2_ interface because of the mismatch between their work functions. The defects in the MoS_2_ QDs are represented as traps having a potential barrier height ϕ_t_. It should be noted that FTO–MoS_2_ contact is Ohmic and MoS_2_–Au contact is a Schottky contact with a barrier height of approximately 0.24 eV. This explains the charging and discharging mechanisms under positive and negative pulses. Under positive bias, the quantum dots get charged by the carriers injected from the FTO to the charge trapping defect states present in the QD layer. These trapped charges have characteristic lifetimes that are specific to the particular traps. Therefore, when the pulse is off, these charges begin to decay. A series of pulses pump up the traps, causing the PPF observed. On the other hand, voltage pulses with opposite polarity cause the expulsion of the trapped charges from the traps, prior to their characteristic timescales, and thus causes depression phenomenon shown in Fig. 3b. This charging and discharging will be kept on happening until the saturation reaches. Since the Poole–Frenkel conduction mechanism and hopping conduction mechanism are temperature dependent, a dedicated study is needed to extract the temperature dependence of PPF/PPD, which is beyond the scope of this work.

## Conclusion

In summary, we have developed a memory concept based on ultra-fine MoS_2_ QD synthesized by liquid-phase exfoliation as an active layer. Memory effects were confirmed with the data retention and endurance measurements using the device structure FTO/MoS_2_/Al without any insulating or capping layer for the QDs. The device exhibits nonvolatile bipolar resistive switching with On–Off ratio of ~ 10^4^_._ The read voltage is found to be of the order of 0.5 V for these devices, which is the lowest reported value for QD based memory devices. We introduced e-synapse based on MoS_2_ QDs for future neuromorphic applications. Further, we have demonstrated Short Term Plasticity in our MoS_2_ QD e-synapse. The e-synaptic behavior is achieved by the charge trapping and de-trapping in the quantum dots rather than the conventional filament-based conduction in memristors. We have performed essential functions of an e-synapse such as PPF and PPD measurement in our e-synapse and found that MoS_2_ QD-based e-synapse can function as a bandpass filter for low-frequency applications. The simplicity in fabrication and excellent memory characteristics suggest that MoS_2_ QDs have potential applications in future storage devices and neuromorphic technology.

### Experimental methods

A simple liquid-phase exfoliation method was employed for the synthesis of MoS_2_ QDs. 250 mg of MoS_2_ powder (< 2 µm size) was sonicated with 25 ml of Dimethylformamide (DMF) solution using a probe-sonicator to mechanically exfoliate MoS_2_ powder. Then the dispersion was continuously stirred for 6 h at 140 °C. After that, the suspension was centrifuged to separate the sediment and supernatant. The resulting light-yellow supernatant was collected and vacuum dried to remove the excess solvent. For further characterization, the residue was dispersed again in water. The MoS_2_ QDs films were fabricated on fluorine-doped tin oxide (FTO) substrate by spin coating. The film was spun at 1,000 rpm for 60 s, followed by annealing at 75 °C for 10 min. Further, aluminum (Al, for ReRAM devices) or gold (Au, for neuromorphic devices) was thermally evaporated on MoS_2_ QD film through a shadow mask to form the top electrodes. This yielded devices of 1 mm^2^ area with 1 mm pitch length.

The bandgap of the MoS_2_ quantum dots was estimated using a UV–Visible absorption spectrometer (Cary 100 Bio UV spectrometer). Raman spectra of the molecular vibrational levels were studied using Raman spectrometer (HORIBA Scientific-Jobin Yvon Technology) and the fluorescent measurements were recorded using a photoluminescence spectrometer (Horiba Scientific Fluoro Max-4 spectrometer). Transmission electron micrographs were taken using a JEOL JEM 2100 high-resolution electron microscope, at an acceleration voltage of 200 kV. The STM imaging and measurements are done in nanoREV STM (Quazar Technology) in constant current mode, with a tip-sample bias of − 0.5 V at a tunneling current of 0.5nA.

## Supplementary information

Supplementary information 1.
