# Supplementary material for: Programmable electronic synapse and nonvolatile resistive switches using MoS2 quantum dots
Source: Sci Rep. 2020 Jul 24;10:12450. doi: 10.1038/s41598-020-68822-5 (PMC7381601; doi:10.1038/s41598-020-68822-5)
Supplement: Supplementary file 1 — Supplementary information 1. [file 41598_2020_68822_MOESM1_ESM.docx]

**Supplementary Information**

**Programmable electronic synapse and nonvolatile resistive switches using MoS2 quantum dots**

Anna Thomas, A.N. Resmi, Akash Ganguly, K.B. Jinesh

Department of Physics, Indian Institute of Space-Science and Technology (IIST), Valiyamala, Thiruvananthapuram – 695547, Kerala, India.


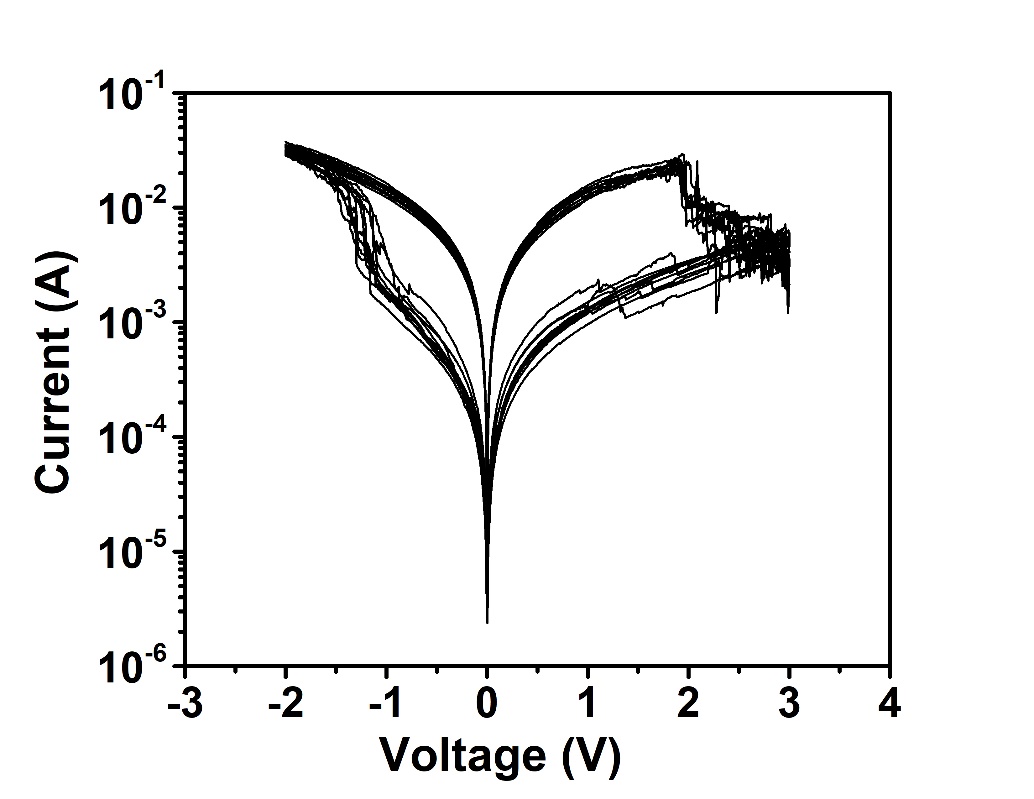


**Figure S1**:Repeated cycling of MoS_2_ QD memory device under ambient condition for 60 cycles.


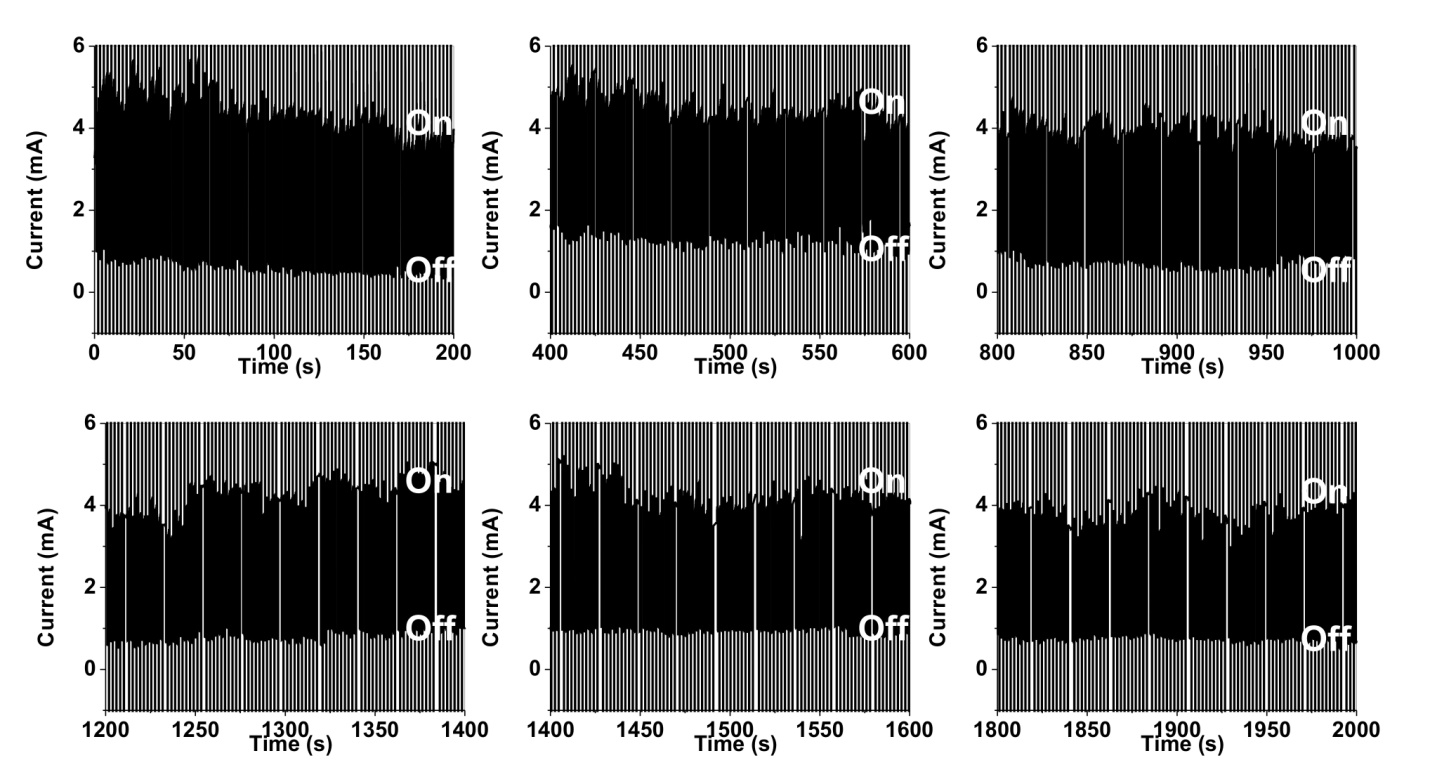


**Figure S2**: The write-read-erase-read cycling of the device at pulse voltages of -1.9, 0.5, and 2.2V respectively for 2000 second measurements. The graphs are shown in six windows, but the measurement data is continuous. The enlarged version of the data is shown in Figure 2(e) in the manuscript.


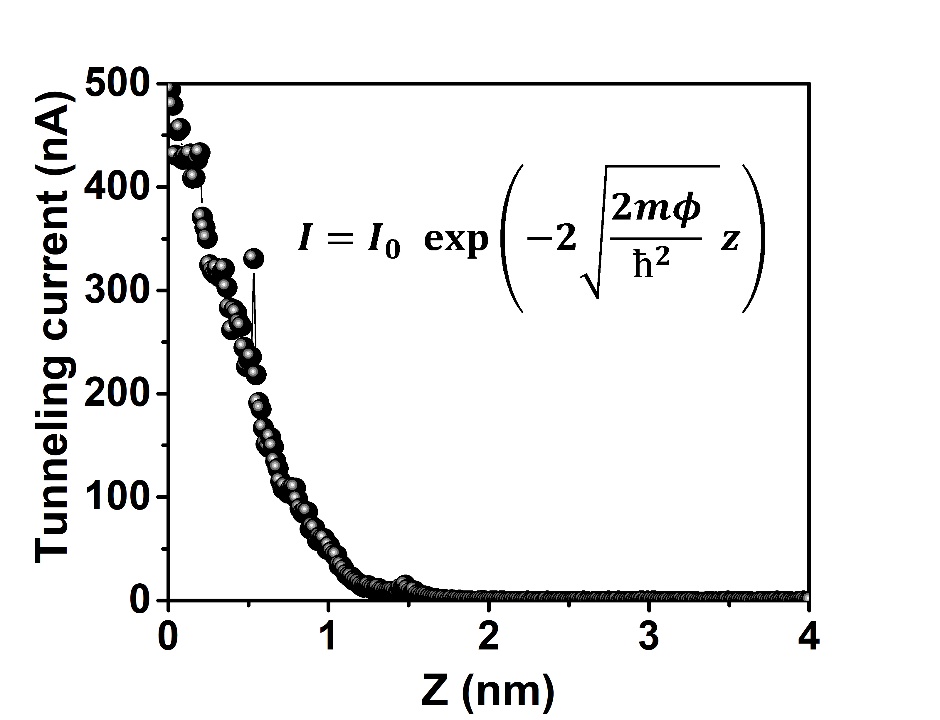


**Figure S3:**  I-z spectrum of MoS_2_ quantum dots measured using a scanning tunneling microscope (STM).

The local work function has been calculated from I-Z (z is the Tip –sample distance) measurement. Tunnelling current exponentially decays with tunnel gap ie, the distance between tip and sample, it is given in the equation 1. From the slope of the I-Z spectra we can extract the workfunction using equation 2. Since, the extracted workfunction is related to the tip and sample workfunction and the applied bias is given by equation 3. Were $\phi_{s}$ is the sample workfunction, $\phi_{t}$is the tip work function and V is the applied bias voltage.

$$I=I_{0}\exp\left( -2\sqrt{\frac{2m\phi}{ћ^{2}}}z \right) \ldots\ldots\ldots\ldots\ldots\ldots\ldots..1$$

$$\phi=\frac{h^{2}}{8m^{*}\pi^{2}}\left[ \frac{d\left( \ln I_{z} \right)}{dz} \right]^{2}\ldots\ldots\ldots\ldots\ldots\ldots\ldots\ldots\ldots\ldots.2$$

$$\phi=\frac{\phi_{s}+\phi_{t}+eV}{2}\ldots\ldots\ldots\ldots\ldots\ldots\ldots\ldots\ldots3$$

The average work function platinum iridium tip is ~ 5.4eV.
